# Tracking Proliferative History in Lymphocyte Development with Cre-Mediated Sister Chromatid Recombination

**DOI:** 10.1371/journal.pgen.1003887

**Published:** 2013-10-31

**Authors:** Baojun Zhang, Meifang Dai, Qi-Jing Li, Yuan Zhuang

**Affiliations:** Department of Immunology, Duke University Medical Center, Durham, North Carolina, United States of America; The Jackson Laboratory, United States of America

## Abstract

Tracking and isolating live cells based on their proliferative history in live animals remains a technical challenge in animal studies. We have designed a genetic marking system for tracking the proliferative frequency and history of lymphocytes during their development and homeostatic maintenance. This system is based on activation of a fluorescent marker after Cre-dependent recombination between sister chromatids at a specially designed tandem loxP site, named Tlox. We have demonstrated the utility of the Tlox system in tracking proliferative windows of B and T lymphocyte development. We have further applied the Tlox system in the analysis of the proliferative behavior and homeostatic maintenance of Vγ1.1 positive γδ T cells. Our data show that Vγ1.1 T cells generated in neonatal but not adult life are able to expand in the thymus. The expanded Vγ1.1 T cells are preferentially maintained in the liver but not in lymphoid organs. It has been shown that numbers of Vγ1.1 T cells were dramatically increased in the lymphoid organs of *Id3* deficient mice. By combining BrdU and Tlox assays we show that this phenotype is primarily due to enhanced neonatal expansion and subsequent retention of Vγ1.1 T cells. Thus, the Tlox system provides a new genetic tool to track clonal expansion within a defined cell population or tissue type in live animals.

## Author Summary

Identification and isolation of live cells based on their proliferative history remains a technical challenge in genetic analysis of animal models. We have designed a novel genetic tool for tracking dividing cells in live animals. The experimental system is based on a fluorescent reporter, whose expression requires both the activity of Cre recombinase and genome replication. We have successfully tested the reporter system in developing lymphocytes and revealed a unique phenomenon of population expansion involving the innate γδ T lymphocytes generated in neonatal life. The experimental system is adaptable to the analysis of any tissue types when combined with appropriate Cre drivers. It provides a new tool for tracking clonal expansion associated with tissue regeneration or neoplastic growth during the normal life span of animals.

## Introduction

Cell proliferation is a tightly regulated process in tissue development and maintenance of tissue functions. Knowing the frequency and history of cell division is not only important in the study of normal tissue development but also in the investigation of tissue regeneration and tumorogenesis. The most commonly used lineage tracking methods are based on Cre mediated activation of reporters in progenitor cells [1]–[3]. By restricting Cre activity to the progenitor cells, this method is highly effective in tracking clonal expansion of the labeled progenitors [3]. However, reporter activation is not linked to cell cycle and thus alone cannot be used to report the proliferative status of the progenitor population. Thus far, methods available for tracking cell proliferation in live animals are still limited and incompatible with recovery of live cells for subsequent analysis. The most commonly used methods for tracking cell proliferation are based on either incorporation of a nucleotide analog such as bromodeoxyurodine (BrdU) or tritiated thymidine during DNA replication [4] or natural dilution of a genetically activated protein marker such as GFP [5]. For example, BrdU pulse labeling method has been successfully used to define the proliferative windows in thymic T cell development [6]. The BrdU method is simple and generally applicable to all tissues, but the detection of chemical labels is incompatible with retrieval of live cells for further studies. In contrast, detection of GFP expression can be carried out with live cells and therefore has the potential to be combined with additional functional assays. For example, GFP intensity has been used to trace the age of naïve T cells based on the dilution of temporarily activated GFP signals associated with homeostatic proliferation of peripheral T cells [7]. However, for cells that have undergone extensive rounds of proliferation, like lymphocytes during antigen responses or cancer stem cells coming out of a dormant phase, they will lose all GFP signals and become indistinguishable from unlabeled cells in the background.

Cre-mediated mitotic recombination provides another way to permit genetic marking of proliferation events in live mice [8]. In this case, activation of a reporter is strictly dependent on mitotic recombination between homologous chromosomes. This system has been successfully used in labeling and tracking progenitor cells that give rise to tumors [9]. However, the overall recombination efficiency of Cre-mediated mitotic recombination is below 1% of the proliferating population when tested in a broad range of cell types [8] including lymphocytes [10]. While the method is powerful in mosaic analysis, the low frequency of mitotic recombination makes the system less effective as a generic method to evaluate proliferative status of progenitor populations for most tissue types.

To overcome these limitations and to enable tracking cell cycle in live cells, we have designed a sister chromatid recombination system to directly link cell cycle with permanent activation of a fluorescent protein marker. This system is based on the fact that Cre/lox mediated recombination can occur between sister chromatids during cell cycle [11], [12]. Cre-mediated sister chromatid exchange occurs at a much higher frequency than Cre-mediated mitotic recombination between homologous chromosomes [12], [13]. In our design, a non-equal exchange between the marked sister chromatids produces a fraction of progeny that acquire the fluorescent marker. We have tested the system in both cultured fibroblasts and developing lymphocytes in live mice. The mouse lymphoid system represents one of the best experimental models for understanding normal and abnormal cell proliferation in a living organism. Using mice expressing lymphoid specific Cre, we have shown that permanent activation of the fluorescent marker after Cre-mediated recombination is correlated with the well-defined windows of cell cycle. As a proof of principle, we further applied this newly established cell tracking system in the study of the expansion of γδ T cells induced by deletion of the *Id3* gene.

## Results

**Figure 1:**
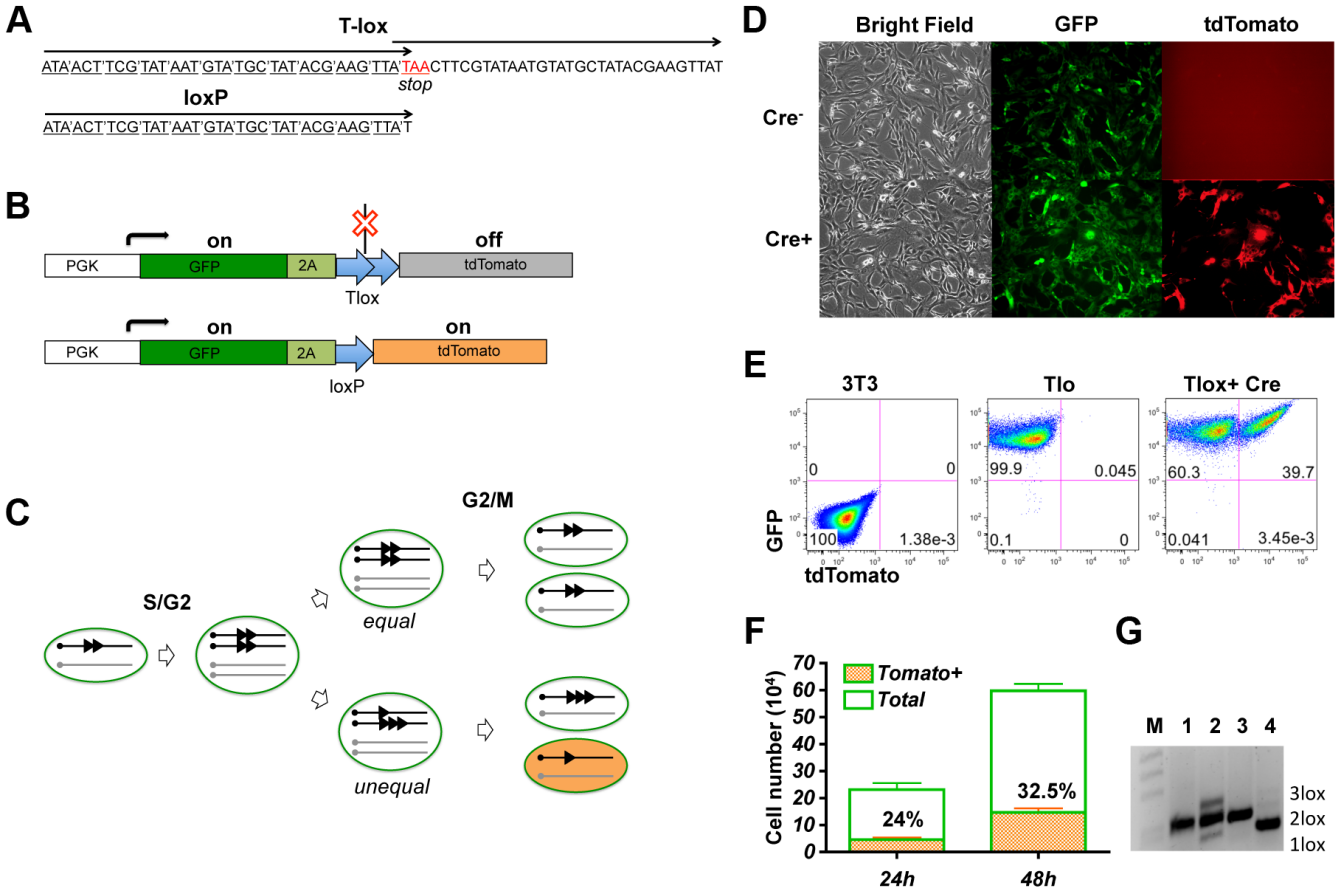
Genetic labeling of proliferating cells *in vitro* with the Tlox design. (A) A comparison between the Tlox and a single loxP sequences. Codons of the reading frame are marked in the Tlox sequence. The stop codon (in red) is in the overlapping region between the first and the second loxP sequences in Tlox. (B) Schematic of Tlox on MSCV retroviral backbone. In the presence of Tlox, both GFP and tdTomato are transcribed, but only GFP (indicated by the green color) is translated. When Tlox is replaced with a single loxP, both GFP and tdTomato (indicated by the orange color) are expressed. 2A is included as a target sequence for auto cleavage [34] to separate GFP and tdTomato. (C) Diagram illustrates Cre-mediated sister chromatid recombination via the Tlox site during mitosis. Results of both equal and unequal exchanges after the G2/M phase are shown. Unequal exchange (bottom part) produces daughter chromosomes carrying either single or triple loxP site. (D–E) 3T3 cells harboring the reporter construct were transduced with control empty virus or Cre virus and analyzed by fluorescent imaging (D) or FACS (E) 48 hours post viral transduction. (F) Cell counts of total and tdTomato positive cells at 24 or 48 hours post viral transduction. Non-transduced cells were excluded based on lack of hCD2 expression. Percentages of tdTomato positive cells are indicated in the plot. (G) PCR assay of genomic DNA with primers flanking the Tlox site. M, 1 kb-DNA size ladder; 1, reporter plasmid control; 2, total cells at 48 hours after Cre transduction; 3, parental cells before Cre transduction; and 4, FACS sorted double positive cells at 48 hours after transduction. Bands representing 1loxP, 2loxP, and 3loxP were verified by sequencing analysis after subcloning the PCR products into Topo vectors.

Cre recombinase has been shown to be capable of driving recombination between sister chromatids via duplicated loxP sites [11], [12]. This recombination system provides an opportunity for Cre mediated activation of a genetic marker during cell cycle. To ensure that Cre/lox mediated recombination occurs exclusively during cell cycle, we have designed a tandem overlapping loxP cassette named Tlox, which contains a stop codon at the beginning of the second loxP unit (Fig. 1A). This Tlox cassette provides a translational stop between the GFP and tdTomato markers (Fig. 1B). The stop codon is eliminated only after the Tlox has been reduced to a single loxP unit. Because recombination requires a Cre tetramer complex [14], intra Tlox recombination cannot occur due to structural constrain. However, Cre-mediated Tlox recombination can occur between two Tlox units present on neighboring sister chromatids during the S/G2/M phase of cell cycle. The recombination will result in either equal or unequal exchange between the sister chromatids (Fig. 1C). While tdTomato remains silent following equal exchange, unequal exchange between sister chromatids should lead to daughter cells inheriting either a single loxP or triple loxP sequences. Whereas cells inheriting a triple loxP remain tdTomato negative, cells inheriting a single loxP should permanently turn on the tdTomato marker. It is anticipated that triple loxP sequences will have additional chances to be converted to a single loxP in subsequent cell cycles through unequal exchange. In principle, this method will label up to one quarter of the progeny from each proliferating cycle if Cre is fully active during S/G2/M phases of each cell cycle. Because the expression of tdTomato is strictly dependent on cell cycle and is permanent, this method offers a new way to identify and isolate cells that have undergone proliferation in the developmental window defined by Cre expression.

We first performed a proof-of-principle test for this genetic design by introducing the Tlox expressing cassette via a retroviral vector into 3T3 cells. As expected, only the GFP signal is detectable in Tlox infected cells in the absence of Cre (Fig. 1D,E). To test the efficiency of sister chromatid recombination, we isolated cells stably expressing GFP and transduced these cells again with a Cre-expressing retroviral vector. Efficiency of transduction was monitored with a hCD2 marker co-expressed with Cre on the retroviral vector [12]. Without cell cycle synchronization, the doubling time for NIH3T3 cells has been reported to be approximately 17 hours [15]. We found 32.5+/−0.5% of Cre transfected cells expressed tdTomato 48 hours post viral transduction, which was up from 24+/−0.7% scored at 24 hours post transduction (Fig. 1F). This number is below the predicted maximal possible frequency (25% for the first cycle and 44% for the second cycle) of tdTomato activation per cell cycle (Fig. 1C). Thus, the labeling frequency observed in real experiments is an underestimation of true proliferation frequency. PCR analysis of the Tlox cassette showed that Cre expression indeed induced generation of both single and triple loxP sequences (Fig. 1G, lane 2). FACS sorted tdTomato positive cells showed exclusively single loxP in the same PCR assay (Fig. 1G, lane 4), supporting the idea that all tdTomato positive cells were the result of Tlox conversion to single loxP.

To introduce a single Tlox cassette into the mouse genome, we used the piggyBac transposon vector [16] to deliver the expression cassette through microinjection of fertilized eggs. FACS analysis of blood samples from founder transgenic mice identified two positive founders (Fig. S1A). We mapped four independent insertion sites through inverse PCR cloning and chose a chromosome 19 insertion (Fig. S1B and C) for subsequent tests.

**Figure 2:**
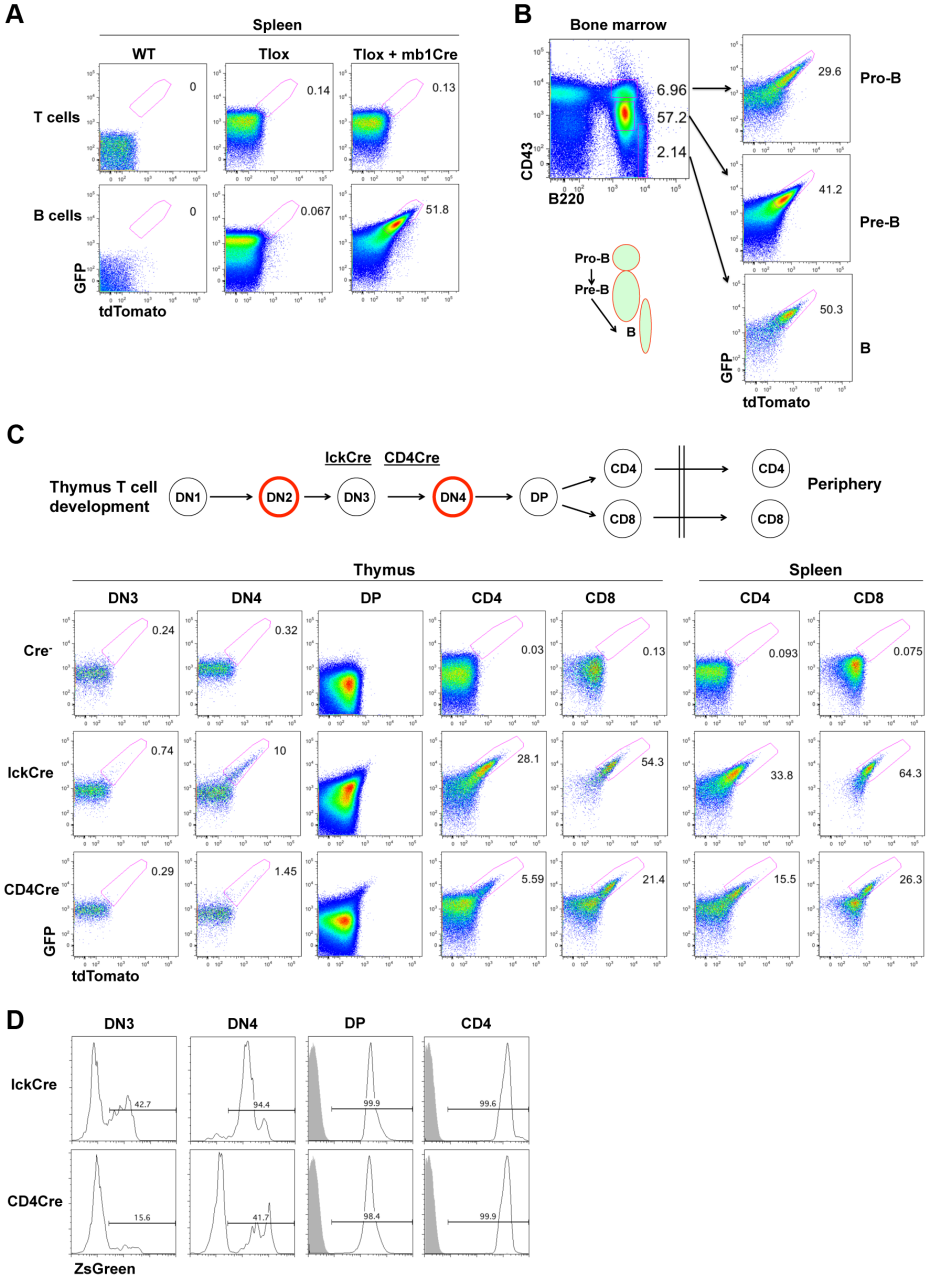
Tlox tracking lymphocyte proliferation *in vivo*. (A) One-month old wild type, Tlox transgenic, Tlox and mb1Cre double transgenic mice were analyzed by gating on live T cells (TCRβ^+^) or B cells (B220^+^) from the spleen. tdTomato positive cells were highlighted by a region gate in the plots. (B) Bone marrow from Tlox and mb1Cre double transgenic mice was analyzed with CD43 and B220 markers. Pro-B, Pre-B and mature B cells were defined as CD43^hi^B220^low^, CD43^med^B220^low^ and CD43^low^B220^hi^, respectively, and are indicated in the FACS plot and the diagram below. Each fraction was analyzed for GFP and tdTomato expression with the tdTomato positive fraction marked in the plot. (C) Diagram and representative FACS plots of major stages of T cell development in the thymus and periphery. Red circle indicates cells undergoing proliferative expansion. The starting time for lckCre and CD4Cre expression around the DN3 stage are indicated by their relative position in the diagram. FACS analyses of tdTomato expression are displayed in sequential order according to stages of T cell development for the Tlox mice (upper panel), the Tlox and lckCre double transgenic mice (middle panel), and the Tlox and CD4Cre double transgenic mice (lower panel). Results are representative of at least three mice for each genotype. DN3 cells are defined as CD4^−^CD8^−^CD44^−^CD25^+^, DN4 cells as CD4^−^CD8^−^CD44^−^CD25^−^, DP cells as CD4^+^CD8^+^, CD4 cells as CD4^+^CD8^−^, and CD8 cells as CD4^−^CD8^+^. DN cells also exclude any CD3, NK1.1, B220 and CD11b positive cells. (D) lckCre (upper panel) and CD4Cre (lower panel) mediated activation of the R26ZsGreen reporter during T cell development. Relative percentage of ZsGreen positive cells at each developmental stage is indicated in each plot. Shaded areas are relevant control populations from ZsGreen mice without a Cre transgene.

The timing and frequency of cell proliferation during lymphocyte development are dynamically regulated during the generation and maintenance of polyclonal lymphocytes. Because the proliferative windows in lymphocyte development have been mapped previously with BrdU labeling methods, we chose lymphocyte development as a test model to validate our Tlox system. We used mb1Cre [17] and LckCre [18] to drive B cell and T cell specific recombination, respectively. mb1Cre initiates Cre expression at the pro-B cell stage and keeps Cre on throughout B cell development. tdTomato expression was not detected in splenic B and T lymphocytes in the absence of mb1Cre, confirming that spontaneous exchanges between sister chromatids are negligible (Fig. 2A, middle column). tdTomato positive cells were exclusively found among B but not T cells in mice carrying both the Tlox transgene and mb1Cre. Activation of tdTomato was coupled with a further upregulation of GFP signals (Fig. S2), which could be a result of increased stability of mRNA after removal of the stop codon in the Tlox cassette and thus eliminating nonsense mediated RNA decay [19]. To further evaluate whether tdTomato expression is linked to cell proliferation, we examined B cell development in the bone marrow. B cell development proceeds in a sequential order from pro-B to pre-B, and then to mature B cells, which are marked as CD43^hi^B220^low^, CD43^med^B220^low^, and CD43^low^B220^hi^ fractions, respectively [20] (Fig. 2B). We found that tdTomato was expressed in all three fractions with the ratio gradually increasing from 30+/−1.9% in pro-B, to 38+/−9.0% in pre-B, and to 43+/−5.3% in mature B cells (Fig. 2B and Fig. S3). The increased frequency of tdTomato expression is correlated with the well-defined pro-B and pre-B windows of cell proliferation [20]. tdTomato expression frequency in mature B was maintained at around 46+/−7.3% in the spleen. The lack of further increase of tdTomato expression in mature B cells from bone marrow to periphery is consistent with their non-cycling state in the absence of antigen stimulation. This result further confirms that Cre cannot drive Tlox recombination in non-cycling cells.

T cell development in the thymus proceeds through several developmental stages with clearly defined windows of proliferation [21]. T lineage commitment and initial expansion of the committed T cell progenitors occur at the DN1 and DN2 stages, respectively. Separation between αβ and γδ T lineage fate is completed at the end of the DN3 stage. Cells choosing the αβ fate expand further at the DN4 stage before entering the non-proliferating DP stage. Clonal selection at the DP stage leads to the formation of either CD4 helper T cells or CD8 cytolytic T cells with each cell expressing a unique TCR. The LckCre transgene has been shown to initiate Cre expression during the DN3 stage of T cell development in the thymus and to support continuing Cre expression thereafter [18], [22] (Fig. 2C). BrdU based analysis has demonstrated that proliferation occurs almost exclusively in the DN2 and DN4 stage of T cell development, although the mature CD8^+^ single positive (SP) T cells have also exhibited a detectable level of proliferation [6], [23]. Mice double positive for the LckCre and the Tlox transgenes were analyzed for tdTomato expression among different cell fractions representing progressive maturation status (Fig. 2C). We found that the earliest stage to detect tdTomato expression was DN4 but not DN3, which is consistent with the idea that LckCre cannot act on the non-proliferating DN3 cells. CD4CD8 double positive (DP) cells are the progeny of rapidly proliferating DN4 cells and have greatly reduced cell size comparing with other immature T cell fractions. Because the overall signal of GFP and tdTomato was dramatically reduced, it was difficult to separate tdTomato positive cells from the negative fractions even though tdTomato expression was clearly visible among DP cells. After progressing to the CD4 or CD8 single positive (SP) stage, cells showed a significantly higher frequency of tdTomato expression than DN4 cells. During the DP to SP transition, immature T cells remain in a non-proliferating state. Therefore, this elevated frequency of tdTomato^+^ SP cells reflected the proliferative transition between DN4 and DP cells. Within the SP populations, the percentage of tdTomato expression in CD8^+^ SP cells is approximately two times higher than that of CD4^+^ SP cells, suggesting additional rounds of cell proliferation have occurred in CD8 lineage cells. This result is consistent with BrdU pulse labeling studies, which also showed higher proliferation rate associated with CD8 SP cells [23]. The frequency of tdTomato expression in both CD4^+^ and CD8^+^ cells showed a moderate increase as they move from the thymus to the spleen. This finding is consistent with the knowledge that naïve T cells rarely proliferate under homeostatic conditions. Overall, the timing and capacity of immature T cell proliferation measured by tdTomato expression from the Tlox cassette are in agreement with the previously defined characteristics of various thymocyte populations during their development.

To gain additional evidence that tdTomato activation is dependent on cell cycle, we compared LckCre-induced with CD4Cre-induced Tlox recombination. CD4Cre is known to support Cre expression starting between the DN3 and DN4 stage [24], which is slightly later than that of LckCre. Correspondingly, the efficiency of tdTomato activation was found lower than that of LckCre at the DN4 stage (Fig. 2C bottom panel). The difference between these two Cre drivers persists even after cells have reached to the CD4 or CD8 stage. This persistent difference cannot be explained by inefficient Cre expression in these later stages since both LckCre and CD4Cre are equally capable of deleting the flox-stop cassette embedded in the R26ZsGreen reporter [25](Fig. 2D), a process independent of cell cycle. Thus, activation of the Tlox reporter requires Cre to be expressed during the proliferative phase of T cell development.

To obtain direct evidence that tdTomato activation is dependent on cell cycle, we performed *in vitro* cell proliferation assay with sorted tdTomato positive or negative CD4 T cells from the Tlox;CD4Cre mice (Fig. S4). TCR-induced cell proliferation was tracked by dilution of the CellTrace Violet dye. tdTomato positive cells retained tdTomato expression during cell cycle (Fig. S4A). tdTomato negative cells showed increased frequency of tdTomato expression after each round of cell cycle (Fig. S4A and B). In fact, tdTomato expression can be detected as early as when cells entering the blasting phase (Fig. S4C). During the same time frame, tdTomato expression was not observed among tdTomato negative cells when they were kept alive without proliferation (Fig. S4B). This study further demonstrates that activation of the Tlox reporter is strictly associated with cycling cells.

**Figure 3:**
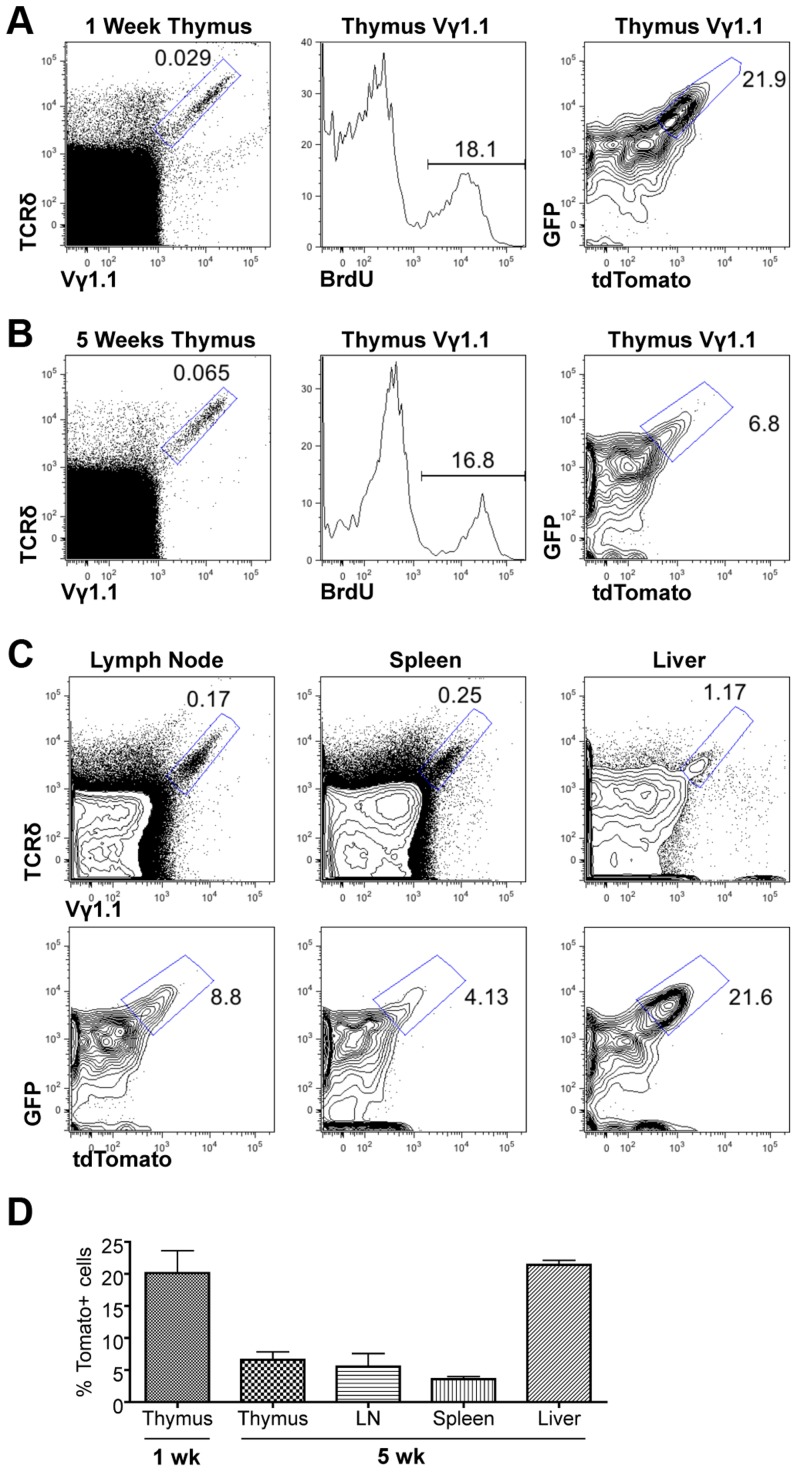
Assessing proliferation frequency and history of Vγ1.1 T cells. (A) Representative FACS plots of thymic Vγ1.1 γδ T cells from one-week old lckCre;Tlox neonates. Vγ1.1 cells were identified in the gated area of TCRδ and Vγ1.1 doubly stained total thymocytes (left) and used in subsequent display of BrdU incorporation (middle) and GFP/dtTomato expression (right). (B) Analyses of 5-week old mice as described in (A). (C) Analysis of Vγ1.1 T cells present in lymph node (left), spleen (middle), and liver (right) of 5-week old mice as described in (B). Frequency of tdTomato expression was shown in the contour plot below each dot plot. (D) Summary of tdTomato expression frequency in Vγ1.1 T cells obtained from (A–C). Sample sizes are three for one-week old and three for 5-week old mice.

We next used the Tlox reporter system to monitor the generation and maintenance of γδ T cells in neonatal and young adult mice. Although majority of γδ T cells found in adult tissues are descendants of fetal derived γδ T cells, γδ T cells are continuously generated in parallel to αβ T cells in postnatal thymus and exported to secondary lymphoid organs [26]. Little is known about the homeostatic maintenance of these post-natal derived γδ T cells. A fraction of γδ T cells enriched for the Vγ1.1 usage (thus referred as Vγ1.1 cells) has been classified as innate-like or NK-like γδ T cells due to their ability to express mixed cytokines including IL4 and γ interferon [27]. These cells are produced in late fetal and neonatal life and detected in the thymus, secondary lymphoid organs, and liver. However, it is not clear whether these cells are continuously generated in postnatal life or maintained exclusively through self-renewal of the fetal-derived population. We first used BrdU pulse labeling method to assess the cell cycle status of γδ T cells in one-week old neonates and five-week old young adult mice (Fig. 3A&B). A similar frequency of BrdU labeling was observed in Vγ1.1 T cells of both age groups. To further evaluate the lineage relationship between the two age groups we used the Tlox assay to evaluate proliferative history of the γδ T cells. The frequency of tdTomato positive Vγ1.1 cells was found significantly higher in one-week old than in 5-week old thymus. While this result is consistent with the earlier studies that Vγ1.1 cells undergo expansion in late fetal and neonatal life [27], the decreased frequency also suggests that Vγ1.1 cells present in young adult thymus are not related to neonatal derived Vγ1.1 T cells. Further analysis of 5-week old mice revealed that Vγ1.1 T cells from other lymphoid organs including lymph nodes and the spleen exhibited a similar low frequency of dtTomato as in the thymus (Fig. 3C&D). In contrast, the frequency of dtTomato positive Vγ1.1 T cells present in the liver is as high as in neonatal thymus. Thus, circulating Vγ1.1 T cells and liver resident Vγ1.1 T cells can be separated into two distinct populations based on their proliferative history.

**Figure 4:**
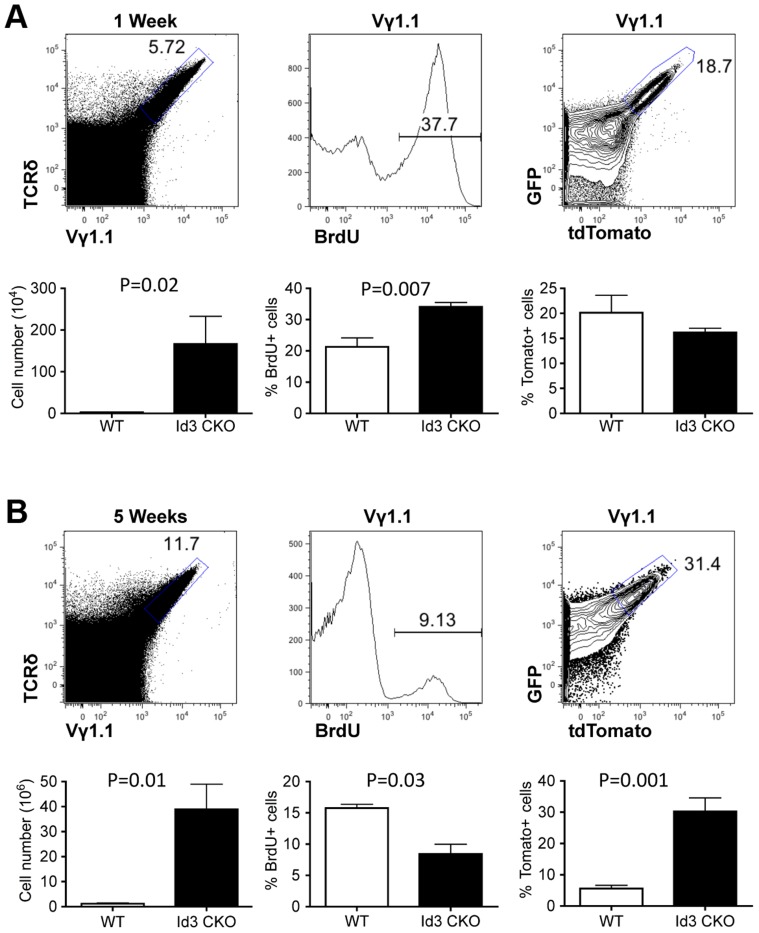
Assessing proliferation frequency and history of Vγ1.1 T cells on *Id3* deficient background. (A) Analysis of Vγ1.1 T cells in one week old LckCre;Tolx;Id3^f/f^ thymus. Vγ1.1 T cells were first gated from analysis of total thymocytes (left) and then displayed for BrdU incorporation (middle) and tdTomato expression (right). Below each FACS plot is statistic analysis against *Id3* wild type samples described in Fig. 3. Four wild type and seven *Id3* mutant samples were used in the unpaired student t-test. (B) Analysis of Vγ1.1 T cells in five week old LckCre;Tolx;Id3^f/f^ thymus. Sample display is as in (A). Student t tests were based on three wild type and three *Id3* mutant samples for the left and center plot and five wild type and six *Id3* mutant samples for the right plot.

Recently, several studies have shown that deletion of the *Id3* gene led to a significant expansion of Vγ1.1 T cells in adult animals [28], [29]. The *Id3* gene encodes a nuclear protein, which regulates lymphocyte development through direct inhibition of E-protein transcription factors [30]. How *Id3* knockout promotes development and/or expansion of Vγ1.1 T cells is still not clear. We thought to further investigate this issue by combining the traditional BrdU method with the newly established Tlox system. LckCre was used to induce T lineage specific deletion of *Id3* and activation of the Tlox marker. We first analyzed cell cycle status with BrdU pulse labeling. A significant increase in BrdU positive cells was observed in *Id3* deficient one-week old neonates in comparison with the wild type controls (Fig. 4A, middle column). However, analysis of young adult mice showed a moderate decrease in BrdU incorporation in *Id3* deficient mice (Fig. 4B, middle column). We then used the Tlox system to track the proliferative history of Vγ1.1 cells. Analysis of Vγ1.1 T cells from one-week old neonates revealed a similar frequency of tdTomato expression between *Id3* deficient and wild type controls (Fig. 4A, right column), suggesting that Vγ1.1 T cells on both backgrounds have gone through similar numbers of cell cycles at the neonatal stage. This result is in contrast to the BrdU data, which detects a higher percentage of proliferating cells in *Id3* deficient Vγ1.1 T cells than in wild type controls within the 4 hour window of pulse labeling. Thus, the proliferation rate revealed by BrdU pulse labeling may not reflect the proliferative history of the cell population. The differential outcomes from these two assays become even more dramatic in the analysis of 5-week old mice. In contrast to the neonates, the frequency of tdTomato positive Vγ1.1 T cells in 5-week old mice was significantly increased in *Id3* deficient mice even though BrdU labeling frequency has decreased (Fig. 4B, middle and right panels). This result supports the idea that *Id3* deficiency promotes development and expansion of Vγ1.1 T cells during neonatal life and their subsequent maintenance in postnatal life.

## Discussion

Our study demonstrated that the Tlox design is an effective genetic tool to track proliferative history of Cre expressing cells in both tissue culture and live animals. This method predicts that the maximal labeling efficiency of dividing cells is 25% per cell cycle (Fig. 1C). While our tracking data clearly shows that Tlox activation is correlated with increased numbers of cell cycle, the overall labeling efficiency is below the expected rate, particularly in animal models. We speculate that multiple factors may contribute to the efficiency of Cre mediated Tlox recombination between sister chromatids. These factors, such as the accessibility of Tlox during S/G2 phase, the expression level of Cre recombinase, and the duration of the S/G2 phase, may vary during development and between tissue types. Thus, the quantitative readout from Tlox assay represents an empirical value associated with the specified developmental system. Once the relative frequency is determined for relevant tissue types in the wild type mice, this reporter assay is particularly useful in assessment of proliferative behavior associated with novel mutations.

When combined with other methods such as BrdU incorporation assays, this system can effectively reveal cell cycle behaviors, some of which would have been otherwise missed or misinterpreted by using the BrdU method alone. Our analysis of developing B cells and αβ T cells confirmed that the frequency of tdTomato expression from the Tlox marker is correlated with Cre expression and windows of cell cycles defined previously by the BrdU method [20], [23], [31]. Using this newly established Tlox assay, we further revealed a change in proliferative behavior of γδ T cells between neonatal and young adult mice. In particular, it has been shown that generation of Vγ1.1 T cells from donor hematopoietic stem cells requires neonatal thymic environment [27]. This observation led to the general hypothesis that Vγ1.1 T cells are produced in late fetal and neonatal life and maintained through self-renewal in postnatal life. Our analysis with the Tlox marker provided strong evidence indicating that most thymic resident Vγ1.1 T cells are not maintained through expansion of preexisting population. They are most likely continuously derived from thymic precursors and quickly turned over in circulation. In contrast to circulating Vγ1.1 T cells, liver resident Vγ1.1 T cells are maintained as a distinct population, which share similar features with Vγ1.1 T cells found in neonatal thymus. Our finding is consistent with the report that liver resident Vγ1.1 T cells lack N addition in their TCR, a feature associated with fetal derived T cells [27]. It remains to be determined whether circulating Vγ1.1 T cells in adult animals can be converted to tissue resident γδ T cells under certain circumstances such as in response to infection or tissue damage. Tlox mediate tracking of expanded populations may assist future investigation of function and homeostatic maintenance of Vγ1.1 T and other lymphoid populations.

*Id3* deficient mice have been characterized to exhibit excess amount of Vγ1.1 T cells in the thymus and peripheral lymphoid organs [28], [29] and develop high incidence of γδ T cell lymphoma at older age [32]. Our study using both BrdU labeling and Tlox tracking methods revealed that *Id3* deletion promotes the development and proliferative expansion of Vγ1.1 T cells in neonatal life. At adult age, this population is apparently maintained by accumulation of cells with a reduced frequency of cell cycle. This result provides a strong evidence to support the finding that Vγ1.1 T cells detected in *Id3* deficient mice exhibit highly restricted TCR usage and often lack N nucleotide addition [29]. Our data support the idea that *Id3* deficiency promotes clonal expansion of Vγ1.1 T cells in the neonatal thymus and, more importantly, their slow expansion and long-term maintenance in adult life. Such a proliferative behavior could contribute to the generation of γδ T cell lymphoma observed in aged *Id3* knockout mice [32]. Our study of *Id3* deficient mice established the Tlox system as a new tool for tracking clonal expansion and possibly for monitoring malignant transformation in live animals.

A major advantage of our Tlox system is the identification and isolation of live cells that have undergone proliferation in a defined window of development. However, additional efforts are still needed for broad applications of this reporter system. Preliminary studies suggest that the Tlox system is inefficiently activated by tamoxifen inducible CreER systems, although the reasons for this and possible steps to optimize efficiency are still under investigation. In addition, the Tlox design can be further adapted to drive expression of other markers or enzymes for easy detection of proliferating cells in tissues other than lymphocytes. It would be particularly attractive to use this method to label and then isolate slowly proliferating somatic stem cells or tumor initiating clones when combined with appropriate Cre drivers. Finally, this recombination system could be combined with live imaging techniques in tracking cell proliferation *in situ* in mice and other model organisms.

## Materials and Methods

### Mice and reagents

The mb1Cre knockin was generated in Dr. Reth's group [17]. The LckCre transgenic [18] and *Id3* conditional knockout [33] alleles were produced in Zhuang lab as previously described. R26ZsGreen strain [25] was purchased from the Jackson Labs. Tlox transgenic lines were produced by microinjection of circular PB donor construct mixed with a helper plasmid (PCX-PBase) at a ratio of 3∶1 [16]. The transgenic procedure was performed by the Duke Transgenic Mouse Facility. Animals were bred and maintained in the SPF facility managed by Duke University Division of Laboratory Animal Research. All animal procedures were approved by the Duke University Institutional Animal Care and Use Committee. The antibodies used were as follows: PE/Cy7 anti-human CD2 (TS1/8), APC/Cy7 anti-mouse TCRβ (H57-597), APC/Cy7 anti-mouse CD4 (GK1.5), PE/Cy7 anti-mouse CD8a (53–6.7), APC anti-mouse B220 (RA2-6B2), PE/Cy7 anti-mouse CD43 Activation-associated Glycoform (1B11), PE/Cy7 anti-mouse/human CD44 (IM7), APC anti-mouse CD25 (3C7), PE/Cy5 anti-mouse NK-1.1(PK136), PE/Cy5 anti-mouse Ly-6G/Ly-6C(Gr-1) (RB6-8C5), PE/Cy5 anti-mouse CD11b(M1/70), APC anti-mouse TCRδ (GL3), PE/Cy5 anti-mouse TCRδ (GL3), APC anti-mouse TCRVγ1.1/Cr4(2.11), PE anti-mouse TCRVγ1.1/Cr4(2.11), APC anti-mouse TCRβ (H57-597) were purchased from Biolegend. The APC BrdU Flow Kit was from BD Biosciences.

### Construction of Tlox retroviral and piggyBac vectors

The PGK promoter was cloned into an MSCV retrovirus backbone. The GFP coding sequence together with a 2A-Tandom loxP was amplified containing multiple cloning sites (Cla I-target cassette-BamH I-Nsi I-Cla I). The fragment of GFP-2A-Tandem loxP was cloned downstream of a PGK promoter using Cla I. The coding sequence of tdTomato was amplified and cloned downstream of the Tlox site using the BamH I-Nsi I linker. For transgenic experiment, the entire GFP-2A-Tlox-tdTomato cassette was subcloned downstream of the Actin promoter present in the piggyBac vector [16].

### Retrovirus and infection

Bosc cells were cultured in complete DMEM medium (containing 10% fetal bovine serum) at 60–70% confluence on 10 cm plates. 10 µg targeting plasmid and 2 µg helper plasmid (pCL-Eco) were transfected into Bosc cells using CaCl_2_. 48 hours later, the supernatant was harvested and filtered with a 0.45 µm-sterile syringe filter. 3T3 cells were infected with virus containing 8 µg/ml polybrene. The medium was changed back to complete DMEM medium 24 hours after infection. GFP^+^ cells were sorted after 2 weeks of subculture. Stable GFP^+^ cells were further infected with either a control virus or Cre-ires-hCD2 virus. FACS analysis and fluorescence imaging were performed at the indicated times.

### FACS analysis and BrdU incorporation assay

Single-cell suspensions were prepared from thymus, spleen, peripheral lymph nodes and bone marrow, and suspended in cold FACS buffer (1×PBS supplemented with 5% bovine calf serum). 1×10^6^ cells were stained with Abs in the dark at 4°C for 30 min. After washing with cold FACS buffer, cell suspensions were analyzed on a FACSCanto II flow cytometer (BD Biosciences). Flowjo software (Tree Star) was used for data analysis. Mice used for BrdU assays were injected i.p. with 1 mg BrdU 4 hours prior to sample collection. BrdU staining was performed according to the manufacture's instruction (BD Biosciences).

## Supporting Information

Figure S1 Identification of Tlox transgenic line. (A) Representative FACS analysis of GFP expression in founder mice derived from co-injection of the PB-Tlox transposon and transposase expressing vector. Blood was drawn from original founders. PBMCs were isolated by Ficoll gradient purification and analyzed for GFP expression on FACS. Representative screening results are shown with two negative and two positive founders. Two out of 32 founders were identified as GFP positive. (B) Diagram of the Tlox integration site on chromosomal 19. Inverse PCR cloning was performed to isolate transposon insertions. Total four independent insertional events were isolated and mapped to four different chromosomes from the two positive funders. These independent insertion events were segregated through subsequent breeding. Insertion event on Chromosome 19 was chosen for its ability to give the highest level of GFP expression. The Tlox cassette was mapped at position 36,354722 mb between the *Andrd1* and *Pcgf5* genes on Chromosome 19. Transcription of GFP and Tomato is in the reverse orientation to the centromere. Mice homozygous for the chromosome 19 insertion allele are phenotypically indistinguishable from wild type littermate controls. Genotyping primers were developed to distinguish the wild type and insertional alleles in a three primer PCR reaction. Primers are as following: TLoxCh19wtF1 5′GTGAGTGATTCATTGGAAGGGACAG (Chr19: 36279890-36279914), TLoxCh19wtR1 5′CTTAATCTCAGTCAGCACCATGCTG (Chr19: 36280707-36280731), RF3 5′CTCGATATACAGACCGATAAAACAC. (C) Representative genotyping results from 6 pups, including three wild type and three Tlox heterozygotes. M: size markers.
(PDF)

Figure S2 Detection of Tomato expression by FACS analysis upon mb1Cre induction. CD19 gated splenic B cells were analyzed for either GFP (A) or tdTomato expression (B). Tlox^+^Cre^+^, Tlox^+^Cre^−^, and Tlox^−^Cre^−^ mice are shown in overlay histograms using the solid line, dotted line, and grey shade, respectively.
(PDF)

Figure S3 Summary of mb1Cre mediated activation of tdTomato in developing B cells. Developing B cells in bone marrow were separated into pro-B, pre-B, and mature B cell stages according to gate defined in Fig. 2B. The percentage of tdTomato positive cells in each fraction is shown with mean and standard error. * indicate p<0.05% based on student t test, N = 3.
(PDF)

Figure S4 *In vitro* proliferation assay of FACS sorted CD4 T cells. (A) Splenic CD4 T cells were sorted from Tlox;CD4Cre^−^ mice (upper panel) or Tlox;CD4Cre^+^ mice. The latter was further separated into Tomato^+^ (middle panel) and Tomato^−^ cells (lower panel). Cells were labeled with CellTrace Violet dye (Invitrogen). 2×10^5^ cells were cultured with 10 ug/ml anti-CD3 Ab and 1 µg/ml anti-CD28 Ab in 96 flat bottom well before analyzed daily for Tomato and Violet signals. (B) Tomato negative CD4 T cells were sorted from Tlox;CD4Cre^+^ mice and labeled with the Violet dye as in (A) before cultured in either non-proliferating condition with the supply of 10 ng/ml of IL-7 or proliferating condition as in (A). The percentage of each gated fraction among total events in the plot is shown next to the plotted areas. The relative percentage of Tomato positive cells within generation 0, 1, and 2 are 16%, 28%, and 30%, respectively, in the Day 2 TCR Stim plot. (C) Forward scatter plot indicates an increase in cell size among Tomato positive cells after one day TCR stimulation of sorted Tomato negative cells.
(PDF)
