# Supplementary figures and images for: Tracking Proliferative History in Lymphocyte Development with Cre-Mediated Sister Chromatid Recombination
Source: PLoS Genet. 2013 Oct 31;9(10):e1003887. doi: 10.1371/journal.pgen.1003887 (PMC3814321; doi:10.1371/journal.pgen.1003887)

**A**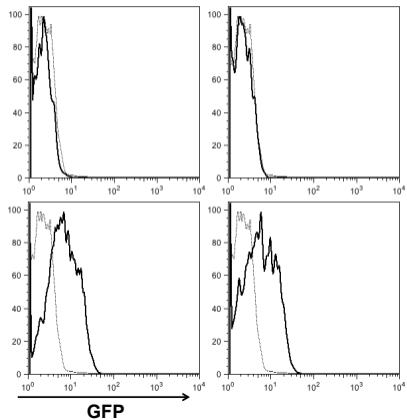**B**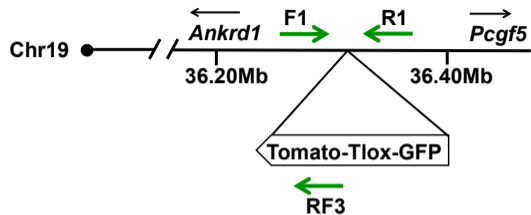**C**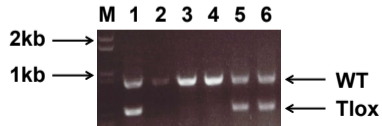

Supplement: Figure S1 — Identification of Tlox transgenic line. (A) Representative FACS analysis of GFP expression in founder mice derived from co-injection of the PB-Tlox transposon and transposase expressing vector. Blood was drawn from original founders. PBMCs were isolated by Ficoll gradient purification and analyzed for GFP expression on FACS. Representative screening results are shown with two negative and two positive founders. Two out of 32 founders were identified as GFP positive. (B) Diagram of the Tlox integration site on chromosomal 19. Inverse PCR cloning was performed to isolate transposon insertions. Total four independent insertional events were isolated and mapped to four different chromosomes from the two positive funders. These independent insertion events were segregated through subsequent breeding. Insertion event on Chromosome 19 was chosen for its ability to give the highest level of GFP expression. The Tlox cassette was mapped at position 36,354722 mb between the Andrd1 and Pcgf5 genes on Chromosome 19. Transcription of GFP and Tomato is in the reverse orientation to the centromere. Mice homozygous for the chromosome 19 insertion allele are phenotypically indistinguishable from wild type littermate controls. Genotyping primers were developed to distinguish the wild type and insertional alleles in a three primer PCR reaction. Primers are as following: TLoxCh19wtF1 5′GTGAGTGATTCATTGGAAGGGACAG (Chr19: 36279890-36279914), TLoxCh19wtR1 5′CTTAATCTCAGTCAGCACCATGCTG (Chr19: 36280707-36280731), RF3 5′CTCGATATACAGACCGATAAAACAC. (C) Representative genotyping results from 6 pups, including three wild type and three Tlox heterozygotes. M: size markers. (PDF) [file pgen.1003887.s001.pdf]

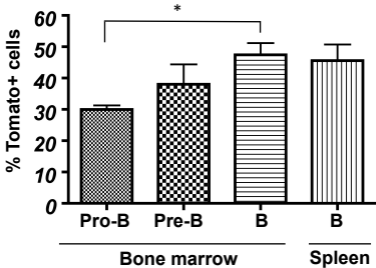

Supplement: Figure S3 — Summary of mb1Cre mediated activation of tdTomato in developing B cells. Developing B cells in bone marrow were separated into pro-B, pre-B, and mature B cell stages according to gate defined in Fig. 2B. The percentage of tdTomato positive cells in each fraction is shown with mean and standard error. * indicate p<0.05% based on student t test, N = 3. (PDF) [file pgen.1003887.s003.pdf]

**A**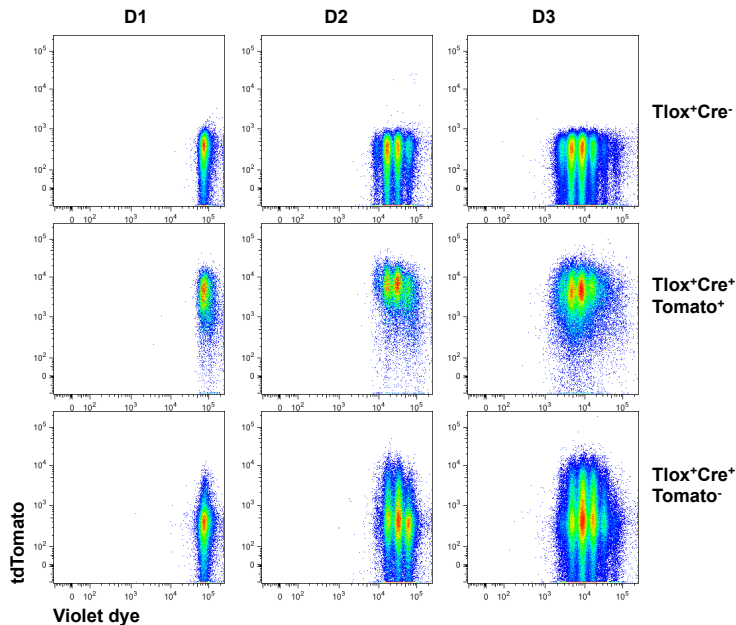**B**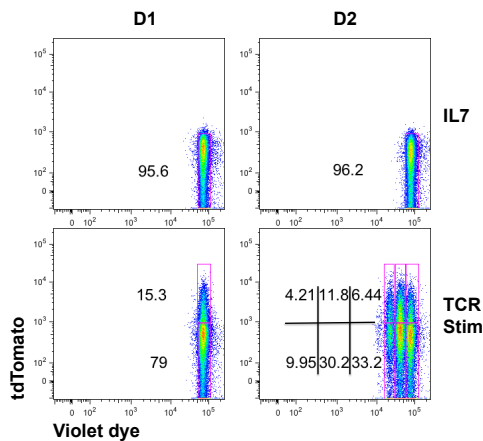**C**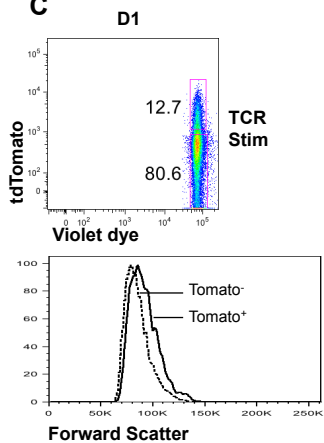

Supplement: Figure S4 — In vitro proliferation assay of FACS sorted CD4 T cells. (A) Splenic CD4 T cells were sorted from Tlox;CD4Cre− mice (upper panel) or Tlox;CD4Cre+ mice. The latter was further separated into Tomato+ (middle panel) and Tomato− cells (lower panel). Cells were labeled with CellTrace Violet dye (Invitrogen). 2×10^5^ cells were cultured with 10 ug/ml anti-CD3 Ab and 1 µg/ml anti-CD28 Ab in 96 flat bottom well before analyzed daily for Tomato and Violet signals. (B) Tomato negative CD4 T cells were sorted from Tlox;CD4Cre+ mice and labeled with the Violet dye as in (A) before cultured in either non-proliferating condition with the supply of 10 ng/ml of IL-7 or proliferating condition as in (A). The percentage of each gated fraction among total events in the plot is shown next to the plotted areas. The relative percentage of Tomato positive cells within generation 0, 1, and 2 are 16%, 28%, and 30%, respectively, in the Day 2 TCR Stim plot. (C) Forward scatter plot indicates an increase in cell size among Tomato positive cells after one day TCR stimulation of sorted Tomato negative cells. (PDF) [file pgen.1003887.s004.pdf]
