# Supplementary material for: Tracking Proliferative History in Lymphocyte Development with Cre-Mediated Sister Chromatid Recombination
Source: PLoS Genet. 2013 Oct 31;9(10):e1003887. doi: 10.1371/journal.pgen.1003887 (PMC3814321; doi:10.1371/journal.pgen.1003887)
Supplement: Figure S2 — Detection of Tomato expression by FACS analysis upon mb1Cre induction. CD19 gated splenic B cells were analyzed for either GFP (A) or tdTomato expression (B). Tlox+Cre+, Tlox+Cre−, and Tlox−Cre− mice are shown in overlay histograms using the solid line, dotted line, and grey shade, respectively. (PDF) [file pgen.1003887.s002.pdf]

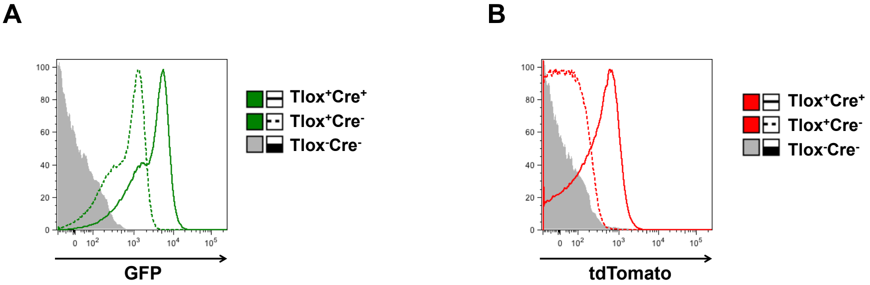
A
B
100
80
60
40
20
0
0
10^2
10^3
10^4
10^5
GFP
tdTomato
Tlox+Cre+
Tlox+Cre-
Tlox-Cre-
